# *N*′-(2,3-Dihy­droxy­benzyl­idene)isonicotinohydrazide

**DOI:** 10.1107/S1600536810048701

**Published:** 2010-11-30

**Authors:** Elif Tecer, Necmi Dege, Ayşin Zülfikaroğlu, Nuray Şenyüz, Hümeyra Batı

**Affiliations:** a Department of Physics, Arts and Sciences Faculty, Ondokuz Mayıs University, 55139 Samsun, Turkey; b Department of Chemistry, Arts and Sciences Faculty, Ondokuz Mayıs University, 55139 Samsun, Turkey

## Abstract

The title compound, C_13_H_11_N_3_O_3_, crystallized with two independent mol­ecules in the asymmetric unit. One of the mol­ecules is twisted while the other is almost planar, with dihedral angles of 28.02 (6) and 2.42 (9)°, respectively, between the benzene and pyridine rings. Intra­molecular O—H⋯O and O—H⋯N hydrogen bonds are present in both mol­ecules. The two independent mol­ecules are linked by pairs of O—H⋯O hydrogen bonds. The crystal structure is further stabilized by inter­molecular N—H⋯N hydrogen bonds and C—H⋯N and C—H⋯O inter­actions.

## Related literature

For the proven therapeutic importance of isonicotinic acid hydrazide, see: Agarwal *et al.* (2005 ▶, 2006 ▶); Savanini *et al.* (2002 ▶). For Schiff base complexes as models for biologically important species, see: Chohan & Sheazi (1999 ▶); Abou-Melha (2008 ▶). For the anti­tubercular activity of hydrazones, see: Durgaprasad & Patel (1973 ▶); Kriza *et al.* (2010 ▶). For hydrogen bonding leading to the dimerization of mol­ecules, see: Avasthi *et al.* (2002 ▶). For delocalized double bonds, see: Zülfikaroğlu *et al.* (2009 ▶). 
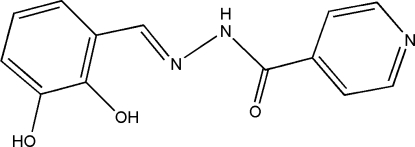


## Experimental

### 

#### Crystal data

C_13_H_11_N_3_O_3_
*M* *_r_* = 257.25
Monoclinic, 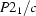

*a* = 7.7781 (2) Å
*b* = 30.0719 (8) Å
*c* = 10.5116 (3) Å
β = 101.551 (2)°
*V* = 2408.89 (11) Å^3^
*Z* = 8
Mo *K*α radiation
μ = 0.10 mm^−1^
*T* = 296 K
0.53 × 0.31 × 0.19 mm

#### Data collection

Stoe IPDS-II diffractometer
Absorption correction: integration (*X-RED32*; Stoe & Cie, 2002 ▶) *T*_min_ = 0.961, *T*_max_ = 0.984
34859 measured reflections
5118 independent reflections
3512 reflections with *I* > 2σ(*I*)
*R*_int_ = 0.049

#### Refinement

*R*[*F* ^2^ > 2σ(*F* ^2^)] = 0.038
*wR*(*F* ^2^) = 0.084
*S* = 1.02
5118 reflections
432 parameters
All H-atom parameters refined
Δρ_max_ = 0.14 e Å^−3^
Δρ_min_ = −0.10 e Å^−3^

### 

Data collection: *X-AREA* (Stoe & Cie, 2002 ▶); cell refinement: *X-AREA*; data reduction: *X-RED32* (Stoe & Cie, 2002 ▶); program(s) used to solve structure: *SHELXS97* (Sheldrick, 2008 ▶); program(s) used to refine structure: *SHELXL97* (Sheldrick, 2008 ▶); molecular graphics: *ORTEP-3* (Burnett & Johnson, 1996 ▶); software used to prepare material for publication: *WinGX* (Farrugia, 1999 ▶) and *PLATON* (Spek, 2009 ▶).

## Supplementary Material

Crystal structure: contains datablocks I, global. DOI: 10.1107/S1600536810048701/su2224sup1.cif

Structure factors: contains datablocks I. DOI: 10.1107/S1600536810048701/su2224Isup2.hkl

Additional supplementary materials:  crystallographic information; 3D view; checkCIF report

## Figures and Tables

**Table 1:** Hydrogen-bond geometry (Å, °)

*D*—H⋯*A*	*D*—H	H⋯*A*	*D*⋯*A*	*D*—H⋯*A*
O1—H1*O*⋯N1	0.96 (2)	1.65 (2)	2.5281 (15)	149.7 (18)
N2—H2*N*⋯N6^i^	0.913 (18)	2.051 (17)	2.9407 (17)	164.7 (15)
O2—H2*O*⋯O1	0.96 (2)	2.20 (2)	2.7022 (14)	111.3 (15)
O2—H2*O*⋯O4	0.96 (2)	1.97 (2)	2.8605 (15)	153.3 (18)
O4—H4*O*⋯N4	0.97 (2)	1.65 (2)	2.5441 (15)	151.2 (19)
N5—H5*N*⋯N3^ii^	0.900 (18)	2.129 (17)	2.9914 (18)	160.2 (14)
O5—H5*O*⋯O1	0.91 (2)	1.96 (2)	2.8138 (16)	154 (2)
O5—H5*O*⋯O4	0.91 (2)	2.25 (2)	2.7171 (16)	111.6 (18)
C10—H10⋯N6^i^	0.964 (17)	2.429 (17)	3.369 (2)	164.8 (15)
C11—H11⋯O6^iii^	0.953 (18)	2.317 (18)	3.2485 (18)	165.4 (13)
C20—H20⋯O2^iv^	0.967 (18)	2.533 (17)	3.4652 (18)	162.0 (13)
C24—H24⋯O3^v^	0.949 (18)	2.395 (18)	3.3373 (18)	172.1 (14)

Symmetry codes: (i) 
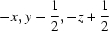
; (ii) 
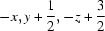
; (iii) 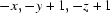; (iv) 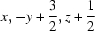; (v) 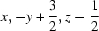.

**Fig. 1.**
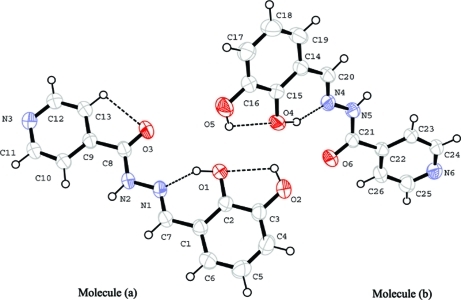
A view of the asymmetric unit of the title compound, showing the molecular structure and atom-numbering scheme of the two independent molecules (a and b). Displacement ellipsoids are drawn at the 50% probability level and H atoms are shown as small spheres of arbitrary radii. The dashed lines show possible hydrogen bonding (see Table 1 for details).

**Fig. 2.**
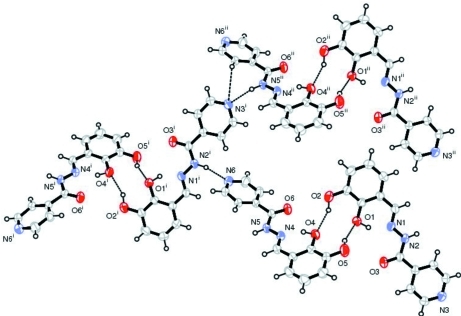
A diagram showing the dimerization of molecules of the title compound through O–H···O, N–H···N and C–H···N hydrogen bonding [Hydrogen bonds are shown as dashed lines - see Table 1 for details; Symmetry codes: (i) -x, 1/2+y, 1/2-z; (ii) x, y, -1+z].

**Fig. 3.**
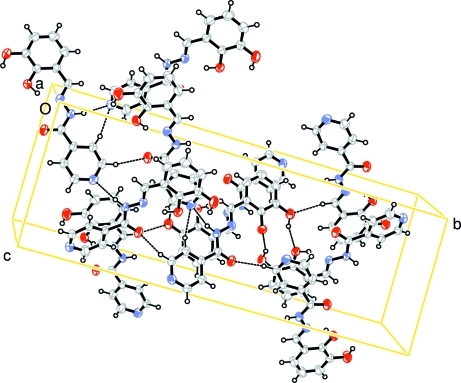
Crystal-packing diagram for the title compound, showing the layered structure formed due to the strong intermolecular hydrogen bonds (dashed lines; see Table 1 for details).

## supplementary crystallographic information

### Comment

Schiff bases are typically formed by the condensation of a primary amine and an aldehyde. Also, Schiff bases are a functional group that contains a carbon-nitrogen (C=N) double bond (an imine group). These bases play an important role in inorganic chemistry as they easily form stable complexes with most transition metal ions. The development of the field of bioinorganic chemistry has increased the interest in Schiff base complexes, since it has been recognized that many of these complexes may serve as models for biologically important species (Abou-Melha 2008; Chohan *et al.*, 1999). The remarkable biological activity of acid hydrazides R—CO—NH—NH_2_, a class of Schiff base, their corresponding aroylhydrazones, R—CO—NH—N=CH—*R*' and the dependence of their mode of chelation with transition metal ions present in the living system have been of significant interest in the past. Isonicotinic acid hydrazide (INH) is a drug of proven therapeutic importance and is used against a wide spectrum of bacterial ailments, *e.g.*, tuberculosis (Agarwal *et al.*, 2005, Savanini *et al.*, 2002). Hydrazones derived from the condensation of isonicotinic acid hydrazide with pyridine aldehydes have been found to show better antitubercular activity than INH (Kriza *et al.*, 2010, Agarwal *et al.*,2006, Durgaprasad *et al.*,1973).

The molecular structure of the two independent molecules (a and b) of the title compound are shown in Fig. 1. The title molecule comprises three functional groups: pyridine, phenyl and hydrazone. Both molecules, a and b, have the *E* configuration at the central C=N bond (Fig. 1). One of the molecules (b) is twisted while the other (a) is planar, with the dihedral angles between the phenyl and pyridine rings being 28.02 (6)° and 2.42 (9)° in molecules (b) and (a), respectively. Because of this different conformation molecules (a) and (b) have different hydrogen bonding patterns. Molecule (a) is influenced by the O—H···N, O—H···O intramolecular hydrogen bonds and C—H···O intramolecular interactions, and the intermolecular N—H···N, O—H···O, C—H···O and C—H···N hydrogen bonds (Table 1). Molecule (b) is influenced by O—H···N and O—H···O intramolecular hydrogen bonds and N—H···N, O—H···O and two C—H···O intermolecular hydrogen bonds (Table 1).

The N1—N2 (molecule a) and N4—N5 (molecule b) bond distances of 1.3726 (15) Å and 1.3679 (16) Å, respectively, are appreciably shorter than a typical N—N single bond, such as that found in free 2,4-dinitrophenylhydrazone, i.e. 1.405 (6) Å; this suggests the existence of a delocalized double bond system (Zülfikaroğlu *et al.*, 2009). The N1═C7 (molecule a) and N4═ C20 (molecule b) bond distances of 1.2782 (18) Å and 1.276 (2) Å, respectively, are typical for a double bond. For both molecules, the central torsion angles are C7—N1—N2—C8 at 177.63 (14)° (molecule a) and C21—N5—N4—C20 at 174.77 (14)° (molecule b). Also, the torsion angles N4—C20—C14—C15 and C23—C22—C21—N5 in the molecule (*b*) are 2.16 (22)° and 22.12 (20)°, respectively.

Interestingly, in the crystal the hydrogen bonding (O2—H2O···O4 and O5—H5O···O1) leads to the dimerization of the molecules (Avasthi *et al.*, 2002) (Table 1), as shown in Fig. 2, and finally to the formation of a layer-like structure (Fig. 3).

### Experimental

Isonicotinic acid hydrazide (1.4 g, 10 mmol) was dissolved in 15 ml of absolute ethanol. To this solution 2,3-dihydroxy benzaldehyde (1.38 g, dissolved in 10 ml absolute ethanol) was added. The reaction mixture was refluxed for 5 h and then cooled to room temperature, giving a clear yellow solution. After keeping the solution in air for 10 days, yellow crystals of the title compound, suitable for X-ray diffraction analysis, were obtained. The crystals were isolated washed with cold absolute ethanol and dried in air. Yield 79%. Anal. Calcd. for C_13_H_11_N_3_O_3_: C 60.70, H 4.31, N 16.33%. Found: C 60.64, H 4.39, N 16.39%. Spectroscopic and other synthetic details are given in the archived CIF.

### Refinement

All the H-atoms were located in difference Fourier maps and were freely refined.

### Crystal data

**Table:**

C_13_H_11_N_3_O_3_	*F*(000) = 1072
*M**_r_* = 257.25	*D*_x_ = 1.419 Mg m^−^^3^
Monoclinic, *P*2_1_/*c*	Mo *K*α radiation, λ = 0.71073 Å
Hall symbol: -P 2ybc	Cell parameters from 29970 reflections
*a* = 7.7781 (2) Å	θ = 1.4–27.3°
*b* = 30.0719 (8) Å	µ = 0.10 mm^−^^1^
*c* = 10.5116 (3) Å	*T* = 296 K
β = 101.551 (2)°	Prism, yellow
*V* = 2408.89 (11) Å^3^	0.53 × 0.31 × 0.19 mm
*Z* = 8	

### Data collection

**Table:**

Stoe IPDS-II diffractometer	5118 independent reflections
Radiation source: fine-focus sealed tube	3512 reflections with *I* > 2σ(*I*)
plane graphite	*R*_int_ = 0.049
w–scan rotation	θ_max_ = 26.8°, θ_min_ = 1.4°
Absorption correction: integration (*X-RED32*; Stoe & Cie, 2002)	*h* = −9→9
*T*_min_ = 0.961, *T*_max_ = 0.984	*k* = −37→37
34859 measured reflections	*l* = −13→13

### Refinement

**Table:**

Refinement on *F*^2^	Secondary atom site location: difference Fourier map
Least-squares matrix: full	Hydrogen site location: inferred from neighbouring sites
*R*[*F*^2^ > 2σ(*F*^2^)] = 0.038	All H-atom parameters refined
*wR*(*F*^2^) = 0.084	*w* = 1/[σ^2^(*F*_o_^2^) + (0.0416*P*)^2^ + 0.005*P*] where *P* = (*F*_o_^2^ + 2*F*_c_^2^)/3
*S* = 1.02	(Δ/σ)_max_ = 0.001
5118 reflections	Δρ_max_ = 0.14 e Å^−^^3^
432 parameters	Δρ_min_ = −0.10 e Å^−^^3^
0 restraints	Extinction correction: *SHELXL97* (Sheldrick, 2008), Fc^*^=kFc[1+0.001xFc^2^λ^3^/sin(2θ)]^-1/4^
Primary atom site location: structure-invariant direct methods	Extinction coefficient: 0.0038 (6)

### Special details

**Table:**

Experimental. Elemental analyses were performed using standard methods at TUBİTAK (The Turkish Scientific Research Centre). The IR spectrum was recorded on a Vertex 80v sample Compartment RT-DLaTGS spectrophotometer operating within 4000–500 cm^-1^. ^1^H NMR spectra were obtained on BRUKER DPX-400, 400 MHz High Performance Digital FT-NMR spectrometer using deuterated as solvent.Spectroscopic characterization of the title compound: The structure was verified by means of IR, ^1^H NMR (DMSO), UV-VIS spectral data and elemental analyses. In the ^1^H NMR spectra of I the azomethine proton appears as a singlet at 8.68 p.p.m.. A single resonance for the proton in –NHN= group is observed at 12.51 p.p.m.. Chemical shifts of the protons on the pyridine ring exhibit two sets of signals in 8.93–8.67 p.p.m. as a doublet and in 8.02–7.74 p.p.m. as doublet, too. The phenyl protons of 1 resonate at 7.07–6.70 p.p.m.. The protons of the phenolic OH are observed at 11.14 p.p.m. and 9.59 p.p.m. as singlets. The IR spectrum of the I shows a weak band at 3520 cm^-1^ assigned to ν OH of the phenolic group. The deformation vibration, δ of the phenolic OH groups appears at 1272 cm^-1^. The NH stretching absorption appears as strong band 3392 cm^-1^. Another important band occurs at 1680 cm^-1^ attributed to ν(C=0) (carbonyl) mode. Azomethine ν(C=N) absorption band appear at 1561 cm^-1^. The medium intensity band at 1061 cm^-1^ is ascribed to ν(N—N) vibration.
Geometry. All e.s.d.'s (except the e.s.d. in the dihedral angle between two l.s. planes) are estimated using the full covariance matrix. The cell e.s.d.'s are taken into account individually in the estimation of e.s.d.'s in distances, angles and torsion angles; correlations between e.s.d.'s in cell parameters are only used when they are defined by crystal symmetry. An approximate (isotropic) treatment of cell e.s.d.'s is used for estimating e.s.d.'s involving l.s. planes.
Refinement. Refinement of *F*^2^ against ALL reflections. The weighted *R*-factor *wR* and goodness of fit *S* are based on *F*^2^, conventional *R*-factors *R* are based on *F*, with *F* set to zero for negative *F*^2^. The threshold expression of *F*^2^ > σ(*F*^2^) is used only for calculating *R*-factors(gt) *etc*. and is not relevant to the choice of reflections for refinement. *R*-factors based on *F*^2^ are statistically about twice as large as those based on *F*, and *R*- factors based on ALL data will be even larger.

### Fractional atomic coordinates and isotropic or equivalent isotropic displacement parameters (Å^2^)

**Table:**

	*x*	*y*	*z*	*U*_iso_*/*U*_eq_	
C1	0.34616 (19)	0.51866 (5)	0.32097 (13)	0.0431 (3)	
C2	0.35373 (19)	0.56269 (5)	0.36519 (13)	0.0419 (3)	
C3	0.4074 (2)	0.59653 (5)	0.29149 (14)	0.0462 (3)	
C4	0.4506 (2)	0.58674 (6)	0.17444 (16)	0.0581 (4)	
C5	0.4439 (3)	0.54342 (6)	0.12981 (17)	0.0640 (5)	
C6	0.3938 (2)	0.50971 (6)	0.20279 (16)	0.0568 (4)	
C7	0.2901 (2)	0.48325 (5)	0.39706 (14)	0.0453 (3)	
C8	0.14430 (19)	0.47313 (4)	0.68980 (14)	0.0444 (3)	
C9	0.08913 (19)	0.43654 (4)	0.77030 (13)	0.0420 (3)	
C10	0.0715 (2)	0.39237 (5)	0.73485 (15)	0.0534 (4)	
C11	0.0135 (2)	0.36246 (5)	0.81626 (15)	0.0540 (4)	
C12	−0.0071 (3)	0.41568 (5)	0.96253 (16)	0.0599 (4)	
C13	0.0503 (2)	0.44791 (5)	0.88844 (15)	0.0547 (4)	
C14	0.4163 (2)	0.72836 (5)	0.73128 (14)	0.0449 (3)	
C15	0.41093 (19)	0.68485 (4)	0.68380 (13)	0.0422 (3)	
C16	0.5024 (2)	0.65111 (5)	0.76002 (14)	0.0480 (4)	
C17	0.5992 (2)	0.66094 (6)	0.88134 (16)	0.0566 (4)	
C18	0.6080 (3)	0.70385 (6)	0.92819 (17)	0.0643 (5)	
C19	0.5175 (2)	0.73716 (6)	0.85442 (16)	0.0585 (4)	
C20	0.3177 (2)	0.76380 (5)	0.65502 (15)	0.0484 (4)	
C21	0.0455 (2)	0.77429 (5)	0.34901 (14)	0.0464 (4)	
C22	−0.0175 (2)	0.81153 (4)	0.25675 (14)	0.0435 (3)	
C23	0.0552 (2)	0.85359 (5)	0.26961 (15)	0.0464 (3)	
C24	−0.0049 (2)	0.88488 (5)	0.17529 (15)	0.0525 (4)	
C25	−0.1971 (3)	0.83591 (6)	0.05928 (17)	0.0614 (4)	
C26	−0.1452 (2)	0.80275 (5)	0.14845 (16)	0.0558 (4)	
N1	0.24130 (16)	0.49326 (4)	0.50259 (11)	0.0446 (3)	
N2	0.18813 (17)	0.46034 (4)	0.57653 (12)	0.0458 (3)	
N3	−0.02774 (18)	0.37320 (4)	0.92838 (12)	0.0526 (3)	
N4	0.22153 (17)	0.75411 (4)	0.54531 (11)	0.0472 (3)	
N5	0.13318 (18)	0.78684 (4)	0.46848 (12)	0.0494 (3)	
N6	−0.12988 (19)	0.87675 (4)	0.07101 (12)	0.0563 (3)	
O1	0.30926 (15)	0.57472 (3)	0.47939 (9)	0.0522 (3)	
O2	0.41509 (17)	0.63953 (3)	0.33438 (11)	0.0598 (3)	
O3	0.14895 (16)	0.51167 (3)	0.72583 (10)	0.0587 (3)	
O4	0.31740 (15)	0.67309 (3)	0.56449 (9)	0.0512 (3)	
O5	0.49762 (18)	0.60853 (4)	0.71623 (12)	0.0666 (3)	
O6	0.02582 (17)	0.73564 (3)	0.31578 (10)	0.0636 (3)	
H1O	0.276 (3)	0.5475 (7)	0.5152 (19)	0.090 (6)*	
H2N	0.180 (2)	0.4320 (6)	0.5448 (15)	0.057 (5)*	
H2O	0.375 (3)	0.6411 (7)	0.415 (2)	0.099 (7)*	
H4	0.486 (2)	0.6103 (6)	0.1235 (15)	0.061 (5)*	
H4O	0.260 (3)	0.7006 (8)	0.531 (2)	0.095 (7)*	
H5	0.472 (2)	0.5371 (6)	0.0478 (18)	0.077 (5)*	
H5N	0.128 (2)	0.8148 (6)	0.4980 (16)	0.059 (5)*	
H5O	0.427 (3)	0.6065 (8)	0.636 (2)	0.106 (8)*	
H6	0.390 (2)	0.4797 (6)	0.1748 (15)	0.060 (5)*	
H7	0.294 (2)	0.4536 (5)	0.3685 (14)	0.051 (4)*	
H10	0.096 (2)	0.3824 (6)	0.6532 (17)	0.066 (5)*	
H11	0.003 (2)	0.3318 (6)	0.7931 (15)	0.061 (5)*	
H12	−0.034 (2)	0.4237 (6)	1.0435 (18)	0.074 (5)*	
H13	0.060 (2)	0.4793 (6)	0.9133 (16)	0.068 (5)*	
H17	0.661 (2)	0.6365 (6)	0.9290 (16)	0.064 (5)*	
H18	0.679 (2)	0.7098 (6)	1.0143 (18)	0.073 (5)*	
H19	0.522 (2)	0.7674 (6)	0.8835 (16)	0.071 (5)*	
H20	0.325 (2)	0.7937 (6)	0.6894 (15)	0.060 (5)*	
H23	0.151 (2)	0.8611 (5)	0.3421 (15)	0.052 (4)*	
H24	0.043 (2)	0.9140 (6)	0.1819 (16)	0.064 (5)*	
H25	−0.286 (3)	0.8311 (6)	−0.0207 (19)	0.082 (6)*	
H26	−0.199 (2)	0.7732 (6)	0.1373 (16)	0.064 (5)*	

### Atomic displacement parameters (Å^2^)

**Table:**

	*U*^11^	*U*^22^	*U*^33^	*U*^12^	*U*^13^	*U*^23^
C1	0.0451 (8)	0.0384 (7)	0.0454 (7)	0.0024 (6)	0.0085 (6)	0.0008 (6)
C2	0.0449 (8)	0.0393 (7)	0.0405 (7)	0.0000 (6)	0.0060 (6)	0.0019 (6)
C3	0.0504 (9)	0.0391 (8)	0.0478 (8)	0.0000 (6)	0.0071 (7)	0.0057 (6)
C4	0.0643 (11)	0.0556 (10)	0.0587 (10)	0.0014 (8)	0.0227 (8)	0.0135 (8)
C5	0.0803 (13)	0.0641 (11)	0.0557 (9)	0.0077 (9)	0.0331 (9)	0.0021 (8)
C6	0.0709 (11)	0.0454 (9)	0.0583 (9)	0.0057 (8)	0.0228 (8)	−0.0051 (7)
C7	0.0523 (9)	0.0330 (8)	0.0504 (8)	0.0006 (6)	0.0096 (7)	−0.0012 (6)
C8	0.0508 (9)	0.0330 (7)	0.0490 (8)	0.0004 (6)	0.0086 (7)	0.0007 (6)
C9	0.0443 (8)	0.0353 (7)	0.0457 (7)	0.0011 (6)	0.0078 (6)	0.0011 (6)
C10	0.0756 (11)	0.0383 (8)	0.0504 (9)	−0.0048 (8)	0.0228 (8)	−0.0033 (7)
C11	0.0726 (11)	0.0340 (8)	0.0569 (9)	−0.0064 (7)	0.0165 (8)	−0.0012 (7)
C12	0.0894 (13)	0.0462 (9)	0.0480 (9)	−0.0041 (9)	0.0228 (9)	−0.0016 (7)
C13	0.0771 (12)	0.0376 (8)	0.0521 (9)	−0.0036 (8)	0.0190 (8)	−0.0030 (7)
C14	0.0509 (9)	0.0400 (8)	0.0460 (8)	−0.0012 (7)	0.0151 (7)	−0.0035 (6)
C15	0.0480 (8)	0.0393 (8)	0.0402 (7)	−0.0011 (6)	0.0113 (6)	−0.0011 (6)
C16	0.0543 (9)	0.0399 (8)	0.0498 (8)	0.0008 (7)	0.0106 (7)	−0.0004 (6)
C17	0.0580 (11)	0.0562 (10)	0.0529 (9)	0.0070 (8)	0.0044 (8)	0.0055 (8)
C18	0.0656 (11)	0.0689 (12)	0.0523 (10)	0.0003 (9)	−0.0028 (9)	−0.0103 (9)
C19	0.0667 (11)	0.0513 (10)	0.0561 (9)	−0.0041 (8)	0.0087 (8)	−0.0145 (8)
C20	0.0622 (10)	0.0353 (8)	0.0509 (9)	0.0000 (7)	0.0188 (8)	−0.0049 (7)
C21	0.0629 (10)	0.0325 (7)	0.0479 (8)	0.0024 (7)	0.0208 (7)	−0.0017 (6)
C22	0.0539 (9)	0.0339 (7)	0.0467 (8)	0.0035 (6)	0.0195 (7)	−0.0021 (6)
C23	0.0599 (10)	0.0353 (8)	0.0463 (8)	0.0003 (7)	0.0165 (8)	−0.0019 (6)
C24	0.0754 (11)	0.0338 (8)	0.0520 (9)	−0.0014 (8)	0.0213 (8)	−0.0002 (7)
C25	0.0696 (12)	0.0520 (10)	0.0585 (10)	−0.0008 (8)	0.0028 (9)	0.0043 (8)
C26	0.0663 (11)	0.0400 (9)	0.0599 (10)	−0.0082 (8)	0.0096 (8)	−0.0003 (7)
N1	0.0524 (7)	0.0340 (6)	0.0469 (7)	−0.0034 (5)	0.0092 (6)	0.0050 (5)
N2	0.0602 (8)	0.0308 (6)	0.0474 (7)	−0.0044 (6)	0.0133 (6)	0.0021 (5)
N3	0.0655 (9)	0.0425 (7)	0.0501 (7)	−0.0038 (6)	0.0123 (6)	0.0053 (6)
N4	0.0638 (8)	0.0335 (6)	0.0472 (7)	0.0073 (6)	0.0179 (6)	0.0023 (5)
N5	0.0726 (9)	0.0303 (6)	0.0463 (7)	0.0102 (6)	0.0141 (6)	0.0002 (5)
N6	0.0732 (9)	0.0437 (7)	0.0533 (8)	0.0070 (7)	0.0156 (7)	0.0053 (6)
O1	0.0784 (8)	0.0367 (6)	0.0438 (5)	−0.0078 (5)	0.0173 (5)	−0.0032 (4)
O2	0.0840 (8)	0.0379 (6)	0.0586 (7)	−0.0085 (5)	0.0171 (6)	0.0042 (5)
O3	0.0850 (8)	0.0314 (5)	0.0626 (6)	−0.0013 (5)	0.0219 (6)	−0.0019 (5)
O4	0.0701 (7)	0.0362 (5)	0.0437 (5)	0.0066 (5)	0.0029 (5)	−0.0044 (4)
O5	0.0877 (9)	0.0389 (6)	0.0653 (8)	0.0103 (6)	−0.0038 (7)	−0.0001 (5)
O6	0.0994 (9)	0.0308 (6)	0.0602 (7)	0.0008 (6)	0.0149 (6)	−0.0044 (5)

### Geometric parameters (Å, °)

**Table:**

C1—C6	1.392 (2)	C15—C16	1.396 (2)
C1—C2	1.4005 (19)	C16—O5	1.3589 (18)
C1—C7	1.450 (2)	C16—C17	1.377 (2)
C2—O1	1.3630 (17)	C17—C18	1.378 (2)
C2—C3	1.3925 (19)	C17—H17	0.961 (18)
C3—O2	1.3669 (18)	C18—C19	1.372 (3)
C3—C4	1.371 (2)	C18—H18	0.979 (19)
C4—C5	1.382 (2)	C19—H19	0.958 (19)
C4—H4	0.961 (17)	C20—N4	1.276 (2)
C5—C6	1.374 (2)	C20—H20	0.967 (17)
C5—H5	0.951 (19)	C21—O6	1.2145 (17)
C6—H6	0.949 (17)	C21—N5	1.3571 (19)
C7—N1	1.2782 (18)	C21—C22	1.498 (2)
C7—H7	0.943 (16)	C22—C26	1.378 (2)
C8—O3	1.2174 (16)	C22—C23	1.381 (2)
C8—N2	1.3582 (18)	C23—C24	1.379 (2)
C8—C9	1.5023 (19)	C23—H23	0.979 (16)
C9—C13	1.379 (2)	C24—N6	1.334 (2)
C9—C10	1.379 (2)	C24—H24	0.950 (18)
C10—C11	1.378 (2)	C25—N6	1.331 (2)
C10—H10	0.964 (17)	C25—C26	1.373 (2)
C11—N3	1.322 (2)	C25—H25	0.99 (2)
C11—H11	0.952 (17)	C26—H26	0.977 (18)
C12—N3	1.328 (2)	N1—N2	1.3726 (15)
C12—C13	1.373 (2)	N2—H2N	0.912 (17)
C12—H12	0.946 (18)	N4—N5	1.3679 (16)
C13—H13	0.977 (18)	N5—H5N	0.901 (17)
C14—C15	1.3981 (19)	O1—H1O	0.96 (2)
C14—C19	1.399 (2)	O2—H2O	0.96 (2)
C14—C20	1.456 (2)	O4—H4O	0.97 (2)
C15—O4	1.3644 (17)	O5—H5O	0.91 (2)
			
C6—C1—C2	118.56 (13)	O5—C16—C15	120.95 (13)
C6—C1—C7	120.89 (14)	C17—C16—C15	119.80 (14)
C2—C1—C7	120.56 (13)	C16—C17—C18	120.71 (16)
O1—C2—C3	116.93 (12)	C16—C17—H17	116.3 (10)
O1—C2—C1	122.82 (12)	C18—C17—H17	123.0 (10)
C3—C2—C1	120.24 (13)	C19—C18—C17	119.92 (16)
O2—C3—C4	119.77 (13)	C19—C18—H18	121.4 (11)
O2—C3—C2	120.41 (13)	C17—C18—H18	118.7 (11)
C4—C3—C2	119.81 (14)	C18—C19—C14	120.99 (16)
C3—C4—C5	120.50 (15)	C18—C19—H19	122.2 (11)
C3—C4—H4	119.4 (10)	C14—C19—H19	116.8 (11)
C5—C4—H4	120.1 (10)	N4—C20—C14	118.63 (13)
C6—C5—C4	120.10 (16)	N4—C20—H20	122.1 (10)
C6—C5—H5	120.2 (11)	C14—C20—H20	119.3 (10)
C4—C5—H5	119.7 (11)	O6—C21—N5	122.91 (14)
C5—C6—C1	120.77 (16)	O6—C21—C22	121.54 (14)
C5—C6—H6	121.7 (10)	N5—C21—C22	115.46 (12)
C1—C6—H6	117.6 (10)	C26—C22—C23	118.00 (14)
N1—C7—C1	118.68 (13)	C26—C22—C21	118.68 (13)
N1—C7—H7	122.3 (9)	C23—C22—C21	123.15 (14)
C1—C7—H7	119.0 (9)	C24—C23—C22	118.79 (15)
O3—C8—N2	123.06 (13)	C24—C23—H23	119.8 (9)
O3—C8—C9	121.07 (13)	C22—C23—H23	121.4 (9)
N2—C8—C9	115.87 (12)	N6—C24—C23	123.50 (15)
C13—C9—C10	117.05 (14)	N6—C24—H24	116.1 (10)
C13—C9—C8	117.67 (13)	C23—C24—H24	120.4 (10)
C10—C9—C8	125.28 (13)	N6—C25—C26	123.41 (17)
C11—C10—C9	119.21 (14)	N6—C25—H25	114.1 (11)
C11—C10—H10	119.9 (10)	C26—C25—H25	122.5 (11)
C9—C10—H10	120.9 (10)	C25—C26—C22	119.33 (16)
N3—C11—C10	124.20 (15)	C25—C26—H26	121.2 (10)
N3—C11—H11	116.2 (10)	C22—C26—H26	119.4 (10)
C10—C11—H11	119.6 (10)	C7—N1—N2	119.80 (12)
N3—C12—C13	124.13 (15)	C8—N2—N1	116.67 (12)
N3—C12—H12	116.8 (11)	C8—N2—H2N	125.0 (10)
C13—C12—H12	119.1 (11)	N1—N2—H2N	118.2 (10)
C12—C13—C9	119.38 (15)	C11—N3—C12	116.00 (13)
C12—C13—H13	123.0 (10)	C20—N4—N5	120.22 (12)
C9—C13—H13	117.5 (10)	C21—N5—N4	116.50 (12)
C15—C14—C19	118.58 (14)	C21—N5—H5N	122.0 (11)
C15—C14—C20	120.91 (13)	N4—N5—H5N	121.4 (11)
C19—C14—C20	120.51 (14)	C25—N6—C24	116.96 (14)
O4—C15—C16	117.12 (12)	C2—O1—H1O	104.8 (12)
O4—C15—C14	122.88 (13)	C3—O2—H2O	109.8 (13)
C16—C15—C14	119.99 (13)	C15—O4—H4O	103.7 (12)
O5—C16—C17	119.26 (14)	C16—O5—H5O	110.2 (15)
			
C6—C1—C2—O1	179.80 (14)	O4—C15—C16—C17	179.97 (14)
C7—C1—C2—O1	−0.3 (2)	C14—C15—C16—C17	−0.7 (2)
C6—C1—C2—C3	0.2 (2)	O5—C16—C17—C18	179.45 (16)
C7—C1—C2—C3	−179.86 (13)	C15—C16—C17—C18	−0.5 (2)
O1—C2—C3—O2	0.5 (2)	C16—C17—C18—C19	1.0 (3)
C1—C2—C3—O2	−179.93 (14)	C17—C18—C19—C14	−0.3 (3)
O1—C2—C3—C4	−178.75 (14)	C15—C14—C19—C18	−0.8 (2)
C1—C2—C3—C4	0.9 (2)	C20—C14—C19—C18	178.64 (16)
O2—C3—C4—C5	179.84 (16)	C15—C14—C20—N4	2.2 (2)
C2—C3—C4—C5	−0.9 (3)	C19—C14—C20—N4	−177.30 (15)
C3—C4—C5—C6	−0.1 (3)	O6—C21—C22—C26	20.5 (2)
C4—C5—C6—C1	1.1 (3)	N5—C21—C22—C26	−162.84 (14)
C2—C1—C6—C5	−1.2 (2)	O6—C21—C22—C23	−154.54 (15)
C7—C1—C6—C5	178.86 (16)	N5—C21—C22—C23	22.1 (2)
C6—C1—C7—N1	−177.40 (15)	C26—C22—C23—C24	1.0 (2)
C2—C1—C7—N1	2.7 (2)	C21—C22—C23—C24	176.10 (14)
O3—C8—C9—C13	−2.3 (2)	C22—C23—C24—N6	−0.3 (2)
N2—C8—C9—C13	177.47 (14)	N6—C25—C26—C22	0.3 (3)
O3—C8—C9—C10	176.64 (16)	C23—C22—C26—C25	−1.0 (2)
N2—C8—C9—C10	−3.5 (2)	C21—C22—C26—C25	−176.33 (15)
C13—C9—C10—C11	1.4 (2)	C1—C7—N1—N2	−179.80 (13)
C8—C9—C10—C11	−177.64 (16)	O3—C8—N2—N1	0.7 (2)
C9—C10—C11—N3	0.1 (3)	C9—C8—N2—N1	−179.09 (12)
N3—C12—C13—C9	0.4 (3)	C7—N1—N2—C8	177.63 (14)
C10—C9—C13—C12	−1.6 (2)	C10—C11—N3—C12	−1.3 (3)
C8—C9—C13—C12	177.49 (16)	C13—C12—N3—C11	1.0 (3)
C19—C14—C15—O4	−179.38 (14)	C14—C20—N4—N5	−177.10 (13)
C20—C14—C15—O4	1.2 (2)	O6—C21—N5—N4	9.1 (2)
C19—C14—C15—C16	1.3 (2)	C22—C21—N5—N4	−167.49 (12)
C20—C14—C15—C16	−178.15 (14)	C20—N4—N5—C21	174.77 (14)
O4—C15—C16—O5	0.0 (2)	C26—C25—N6—C24	0.5 (3)
C14—C15—C16—O5	179.39 (14)	C23—C24—N6—C25	−0.5 (2)

### Hydrogen-bond geometry (Å, °)

**Table:**

*D*—H···*A*	*D*—H	H···*A*	*D*···*A*	*D*—H···*A*
O1—H1O···N1	0.96 (2)	1.65 (2)	2.5281 (15)	149.7 (18)
N2—H2N···N6^i^	0.913 (18)	2.051 (17)	2.9407 (17)	164.7 (15)
O2—H2O···O1	0.96 (2)	2.20 (2)	2.7022 (14)	111.3 (15)
O2—H2O···O4	0.96 (2)	1.97 (2)	2.8605 (15)	153.3 (18)
O4—H4O···N4	0.97 (2)	1.65 (2)	2.5441 (15)	151.2 (19)
N5—H5N···N3^ii^	0.900 (18)	2.129 (17)	2.9914 (18)	160.2 (14)
O5—H5O···O1	0.91 (2)	1.96 (2)	2.8138 (16)	154 (2)
O5—H5O···O4	0.91 (2)	2.25 (2)	2.7171 (16)	111.6 (18)
C10—H10···N6^i^	0.964 (17)	2.429 (17)	3.369 (2)	164.8 (15)
C11—H11···O6^iii^	0.953 (18)	2.317 (18)	3.2485 (18)	165.4 (13)
C20—H20···O2^iv^	0.967 (18)	2.533 (17)	3.4652 (18)	162.0 (13)
C24—H24···O3^v^	0.949 (18)	2.395 (18)	3.3373 (18)	172.1 (14)

Symmetry codes: (i) −*x*, *y*−1/2, −*z*+1/2; (ii) −*x*, *y*+1/2, −*z*+3/2; (iii) −*x*, −*y*+1, −*z*+1; (iv) *x*, −*y*+3/2, *z*+1/2; (v) *x*, −*y*+3/2, *z*−1/2.
